# Isolation and evolution of labile sulfur allotropes *via* kinetic encapsulation in interactive porous networks

**DOI:** 10.1107/S2052252516008423

**Published:** 2016-06-08

**Authors:** Hakuba Kitagawa, Hiroyoshi Ohtsu, Aurora J. Cruz-Cabeza, Masaki Kawano

**Affiliations:** a Division of Advanced Materials Science, Pohang University of Science and Technology, RIST Building 3-3390, 77 Cheongam-Ro, Nam-Gu, Pohang, Republic of Korea; b Department of Chemistry, School of Science, Tokyo Institute of Technology, 2-12-1 Ookayama, Meguro-ku, Tokyo, Japan; c School of Chemical Engineering and Analytical Science, University of Manchester, The Mill, Sackville Street, Manchester M13 9PL, England

**Keywords:** sulfur, kinetic trapping, porous coordination networks, X-ray diffraction, allotropes, metal–organic frameworks, MOFs, coordination polymers, transient chemical species

## Abstract

The isolation and characterization of small sulfur allotropes have long remained unachievable because of their extreme lability. This study reports the first direct observation of disulfur (S_2_) with X-ray crystallography. Sulfur gas was kinetically trapped and frozen into the pores of two Cu-based porous coordination networks containing interactive iodide sites. Stabilization of S_2_ was achieved either through physisorption or chemisorption on iodide anions. One of the networks displayed shape selectivity for linear molecules only, therefore S_2_ was trapped and remained stable within the material at room temperature and higher. In the second network, however, the S_2_ molecules reacted further to produce bent-S_3_ species as the temperature was increased. Following the thermal evolution of the S_2_ species in this network using X-ray diffraction and Raman spectroscopy unveiled the generation of a new reaction intermediate never observed before, the *cyclo*-tri­sulfur dication (*cyclo*-S_3_^2+^). It is envisaged that kinetic guest trapping in interactive crystalline porous networks will be a promising method to investigate transient chemical species.

## Abstract 2

Reported here are the isolation and direct observation of extremely reactive S_2_ and its conversion into bent-S_3_ *via* a *cyclo*-S_3_^2+^ intermediate on interactive sites in porous coordination networks.

## 1. Introduction   

Cryogenic trapping methods, coupled with spectroscopy or crystallography, have been widely used to investigate transient chemical species (Whittle *et al.*, 1954 ▸; Misochko *et al.*, 2003 ▸; Kawano, 2014 ▸; Edman *et al.*, 1999 ▸), but these methods do not always allow the observation of very labile reactive intermediates. To circumvent this problem, we propose encapsulation of transient species in an interactive porous network under non-equilibrium conditions. The kinetic trapping method is widely used in cryogenic trapping (Whittle *et al.*, 1954 ▸; Mück *et al.*, 2012 ▸), although there is no report using porous coordination networks and no direct X-ray observation has yet been achieved. The method using porous co­ordination networks might provide a unique way for *in situ* observation of very labile chemical intermediates and for the following of chemical reactions

Many fascinating guest encapsulation studies have been performed in the past using porous coordination networks (Matsuda, 2013 ▸; Cook *et al.*, 2013 ▸; Kitagawa & Uemura, 2005 ▸; Eddaoudi *et al.*, 2001 ▸; Férey, 2008 ▸; Peterson *et al.*, 2014 ▸; Ohmori *et al.*, 2005 ▸). Most of these studies, however, have only been carried out in conditions of thermodynamic equilibrium, which makes the observation of transient intermediates hardly possible (Kubota *et al.*, 2014 ▸; Ikemoto *et al.*, 2014 ▸; Kawamichi *et al.*, 2009 ▸). Although time-resolved techniques offer attractive alternatives, they generally require the reactions to be reversible (Ohashi, 1998 ▸). Our approach involves the stabilization of transient species *via* the active sites located in the channels of porous coordination networks. Here we report the first direct X-ray observation of extremely reactive S_2_ species and their conversion towards bent-S_3_ *via cyclo*-S_3_^2+^ on an interactive site in a channel of a porous coordination network.

Sulfur has a very rich chemistry, with around 30 allotropes known to date, although the transient nature of the smaller allotropes makes their isolation and characterization very challenging (Peramunage & Licht, 1993 ▸; Evers & Nazar, 2013 ▸; Xin *et al.*, 2012 ▸; Meyer, 1976 ▸; Steudel & Eckert, 2003 ▸; Steudel *et al.*, 2003 ▸).

**Figure 1:**
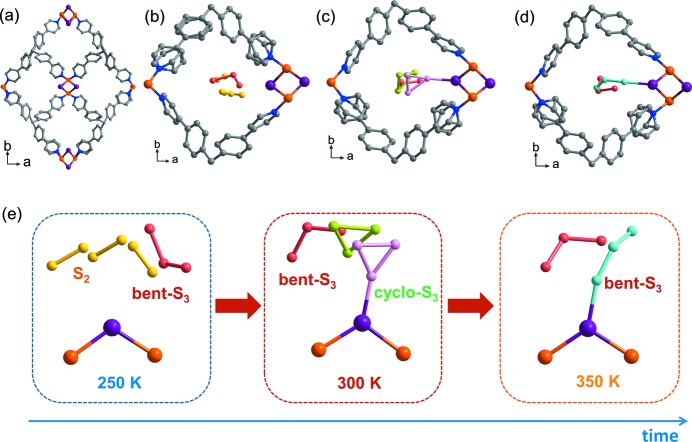
Pore description in the crystal structures of (*a*) network **1**, and (*b*) network **1** after sulfur encapsulation at 250 K, (*c*) 300 K and (*d*) 350 K. (*e*) The crystal structure of sulfur-encapsulating network **1**, showing parts of the {CuI} unit and the sulfur species. (Left) At 250 K, physisorbed S_2_ and bent-S_3_ were observed. (Middle) At 300 K, chemisorbed *cyclo*-S_3_^2+^, physisorbed *cyclo*-S_3_ and bent-S_3_ were observed. (Right) At 350 K, bent-S_3_ was observed. (Bottom) The arrow indicates the time course of the measurements, showing the molecular transformation mechanism from S_2_ to bent-S_3_ species. Atom colouring: Cu orange, I purple, and S yellow, red, green, pink and cyan to distinguish disordered molecules.

In a recent study, we reported the direct observation of bent-S_3_ (tri­sulfur or thio­zone) through encapsulation in a Zn-based porous coordination network (Ohtsu *et al.*, 2013 ▸). The crystal structure of the network–S_3_ complex revealed important interactions between S_3_ and the network iodides of such strength that release of the S_3_ molecules only took place at high temperatures (500 K). We suspect that S_3_ encapsulation might have taken place *via* a ‘ship-in-a-bottle’ type of mechanism (Ichikawa *et al.*, 1991 ▸; Rau *et al.*, 1973 ▸): first the smaller S_2_ (disulfur) enters the pores of the network and then it converts to S_3_ (tri­sulfur) because S_3_ is more stable than S_2_; however, this mechanism remains to be proven. We have also reported the synthesis of two porous coordination networks of CuI with tetra-4-(4-pyridyl)phenylmethane (TPPM) with fascinating properties (Kitagawa *et al.*, 2013 ▸). The kinetic product of the synthesis is network **1** (Fig. 1 ▸ *a*), [(CuI)_2_(TPPM)]*_n_*, which contains molecular-sized channels with accessible iodide sites. These iodide sites are highly interacting and can adsorb molecules such as I_2_ through chemisorption (Kitagawa *et al.*, 2013 ▸). The thermodynamic product of the synthesis is network **2** (Fig. 4*a*), [(Cu_2_I_2_)(TPPM)]*_n_*, which contains smaller one-dimensional channels with no exposed iodide sites. In network **2**, only physisorption of I_2_ is possible within the hydro­phobic one-dimensional channel, because the iodide sites are located in small cavities that are poorly accessible. The channels of networks **1** and **2** have the precise molecular dimensions needed for trapping small molecules, with the added advantage of the interacting iodide sites in network **1**. Network **1** can accommodate S_2_ (5.8 × 3.6 × 3.6 Å) or S_3_ (6.8 × 4.6 × 3.6 Å for the bent form, 5.8 × 5.5 × 3.6 Å for the *cyclo* form) because of its channel size of 5.8 × 5.5 Å. In contrast, network **2** can accommodate only S_2_, because of its channel size of 4.0 × 3.9 Å. Because we expect strong interactions between small sulfur allotropes and the iodide sites of the networks (see the supporting information), these materials may serve as traps for labile sulfur intermediates. In order to isolate the most reactive species, we consciously arrested the encapsulation process before it reached equilibrium *via* cooling (kinetic trapping); we aimed to observe the ‘ship-in-a-bottle’ conversion from reactive S_2_ to the more dynamically stable S_3_ by heating.

## 2. Results and discussion   

### 2.1. Kinetic trapping of sulfur gas   

Sulfur gas was encapsulated in networks **1** and **2** under kinetic conditions; an excess amount of elemental sulfur and desolvated network **1** or **2** were placed at different sites of a zigzag shaped glass tube (see Fig. S1 in the supporting information). The glass tube was then sealed in a vacuum (∼10^−6^ Torr; 1 Torr = 133.322 Pa) and heated in a flame at the site containing the sulfur. The zigzag tube was sufficiently long, and the sulfur and the network thus sufficiently separated, that high temperatures could be reached at the sulfur site while the network was kept at room temperature, creating a sharp temperature gradient. Shortly after heating the sulfur powder, the yellow crystals of network **1** turned dark yellow, whereas the crystals of network **2** did not display a colour change.

### 2.2. Direct observation of transient small sulfur by X-ray diffraction   

Within 5 min of the colour change, a single crystal of network **1** was mounted on a goniometer and X-ray diffraction data were collected at 250 K. Because diffuse scattering was observed at 250 K (see Fig. S4 in the supporting information), the crystal structure was solved making use of Bragg diffractions only (see the supporting information). On the basis of a Laue check, and after careful consideration of various crystal systems and space groups, the structure was solved in the tetragonal 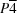 space group.

The crystal structure analysis clearly revealed the existence of physisorbed S_2_ and bent-S_3_ species on the iodide sites of the framework channels (Fig. 1 ▸; see Fig. S5 in the supporting information for structure details). The geometry of S_3_ was found to be in good agreement with that previously reported for [(ZnI_2_)_3_(TPT)_2_(S_3_)]*_n_* by structure solution from X-ray powder diffraction (Ohtsu *et al.*, 2013 ▸) and that of S_3_ in the gas phase as observed by rotational spectroscopy (McCarthy *et al.*, 2004 ▸). These physisorbed S_2_ and bent-S_3_ do not have any interaction with iodide; reactive S_2_ can take part in subsequent reactions because it is not stabilized by the pores.

**Figure 2:**
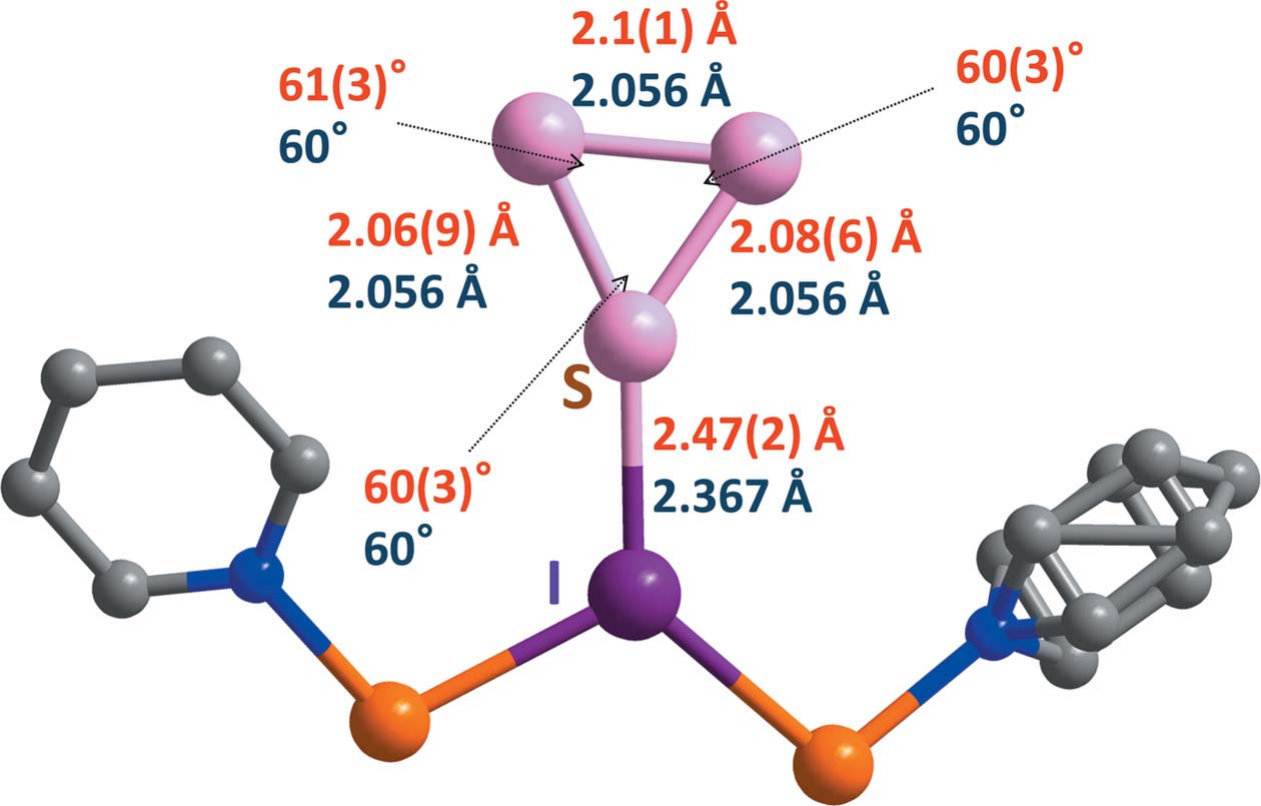
Geometric parameters from X-ray diffraction and theoretical calculation for chemisorbed *cyclo*-S_3_^2+^. Red numbers indicate values obtained from X-ray analysis and blue numbers refer to values obtained from calculation of I—*cyclo*-S_3_^2+^. Atom colouring: Cu orange, I purple and S pink.

In order to investigate the transient nature of S_2_ in the channels of network **1**, we collected two additional sets of X-ray single-crystal diffraction data at 300 and 350 K using a heating rate of 10 K min^−1^ between measurements (see Fig. S3 in the supporting information). The diffraction data at 300 K showed a space-group change from 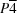 to 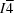, a sharpening of the diffraction spots and the almost complete disappearance of diffuse scattering, which indicates that successive reaction of the sulfur species had taken place on heating. Analysis of the 300 K structure revealed the formation of *cyclo*-S_3_ chemisorbed on bridging iodide sites, and the presence of physisorbed bent-S_3_ and physisorbed *cyclo*-S_3_ in the network **1** channels (Fig. 1 ▸). The *cyclo*-S_3_ allotrope has been predicted to be less stable than the bent-S_3_ structure, but still energetically accessible, by theoretical calculations (Flemmig *et al.*, 2005 ▸) but it had never been observed before. A theoretical investigation of the adsorption of *cyclo*-S_3_ on the network iodide sites revealed that chemisorption is only possible if *cyclo*-S_3_ is present as a dication, *cyclo*-S_3_^2+^ (see the supporting information). Even though we did not use any restraints for the bond lengths, the geometric parameters obtained from this X-ray analysis matched those obtained by theoretical calculation (Fig. 2 ▸). The *cyclo*-S_3_^2+^ state is isoelectric with a *cyclo*-SiS_2_ molecule (Mück *et al.*, 2012 ▸) isolated by matrix isolation, indicating the potential existence of a cyclic form. We could not determine the counter pair formed by oxidation, because of severe disorder of the physisorbed species for which restraints on bond length were used during the refinement. A structure redetermination of the single crystal at an even higher temperature, 350 K, revealed only bent-S_3_ species in network **1**, suggesting a complete transformation of chemisorbed *cyclo*-S_3_^2+^ (and physisorbed *cyclo*-S_3_ species) to bent-S_3_ species (see Fig. S6 in the supporting information). After the heating cycle, the same single crystal was cooled back to 250 K for a second structure redetermination at low temperature, but the diffraction data were not of sufficient quality to allow structure solution. Refinement using the initial 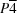 space group was unsuccessful, which indicates an irreversible 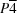 to 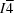 phase transformation.

From this series of X-ray diffraction experiments of sulfur-encapsulating network **1**, we propose one of the possible reaction pathways of small sulfur allotropes: first, S_2_ was kinetically trapped by physisorption and partly transformed into physisorbed bent-S_3_; second, on heating the S_2_ converted to chemisorbed *cyclo*-S_3_^2+^, and physisorbed *cyclo*-S_3_ and bent-S_3_ species; and third, the *cyclo*-S_3_ species transformed to the more stable bent-S_3_ species (Fig. 1 ▸ *e*). Despite the kinetic nature of these experiments, we always found consistent results upon repetition of the diffraction measurements on different single crystals. We also observed chemisorbed S_2_ molecules on the interactive iodide sites (see Fig. S7 in the supporting information). Our theoretical calculations predicted chemisorption of S_2_ to be less favourable than physisorption, because S_2_ needs to change its electronic spin (see the supporting information).

### 2.3. Spectroscopic confirmation of sulfur species   

**Figure 3:**
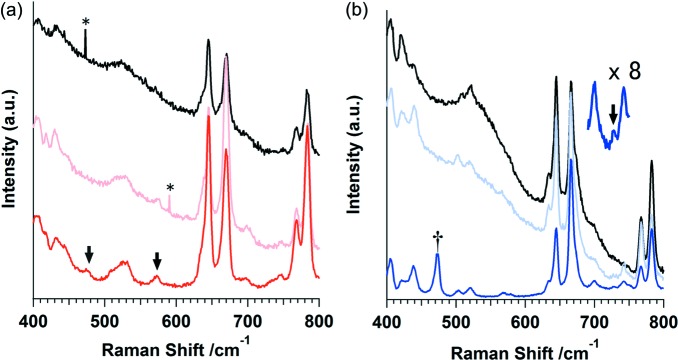
(*a*) Raman spectra of network **1**, desolvated (black), solvated with DMSO (pink) and after sulfur encapsulation (red). (*b*) Raman spectra of network **2**, desolvated (black), solvated with DMSO (pale blue) and after sulfur encapsulation (blue). The inner graph in part (*b*) shows a magnified view of the 690–750 cm^−1^ region for the sulfur-encapsulated network **2** sample. Black arrows highlight the bands appearing after sulfur encapsulation [475 and 573 cm^−1^ for *cyclo*-S_3_^2+^ and bent-S_3_ in part (*a*), and 728 cm^−1^ for S_2_ in part (*b*)]. The asterisks (*) indicate the effects of cosmic rays and the dagger (†) shows *cyclo*-S_8_ on the crystal surface.

The trapping of sulfur in network **1** was also investigated at room temperature using microscopic Raman and IR spectroscopy. Raman spectra of the samples after sulfur encapsulation displayed new bands at 475 and 573 cm^−1^ (Fig. 3 ▸ *a*). These bands have been assigned to chemisorbed *cyclo*-S_3_^2+^ (475 cm^−1^) and chemisorbed *cyclo*-S_3_^2+^ plus bent-S_3_ species (573 cm^−1^) with the help of density functional theory (DFT) calculations (see the supporting information) (Picquenard *et al.*, 1993 ▸). After 18 h, the intensity of the *cyclo*-S_3_^2+^ species band decreased significantly, which suggests that the *cyclo*-S_3_^2+^ species were consumed and converted into bent-S_3_ (see Fig. S9 in the supporting information). Bent-S_3_ species were clearly detected by IR spectroscopy (band at ∼680 cm^−1^; see Fig. S10 in the supporting information). There are two possibilities for the mechanism of the transformation of S_2_ to *cyclo*-S_3_^2+^ to bent-S_3_: (i) direct conversion of S_2_ to S_3_ using catalytic iodide sites; or (ii) conversion including dimethylsulfoxide (DMSO) (see the supporting information). The oxidation of S_3_ into *cyclo*-S_3_^2+^ might be preceded by other sulfur species accepting electrons and protons, resulting in H_2_S_*n*_ species. We observed new bands in the IR spectra in the region of 2225 cm^−1^, which are most likely due to S—H stretches (see Fig. S17 in the supporting information) (Marsden & Smith, 1988 ▸). Attempts to reveal the reaction mechanism by removing DMSO completely resulted in deterioration of the single crystals. A possible reaction mechanism is outlined in the supporting information. However, the reactions occurring are complex and, unless the intermediates are strongly adsorbed on the network (like the species identified by X-ray diffraction), they are difficult to characterize. In fact, although it is not trivial to reveal the reaction mechanism, we clearly observed the structural change in these small sulfur species on an interactive site using X-ray diffraction.

### 2.4. Sulfur species in network 2   

**Figure 4:**
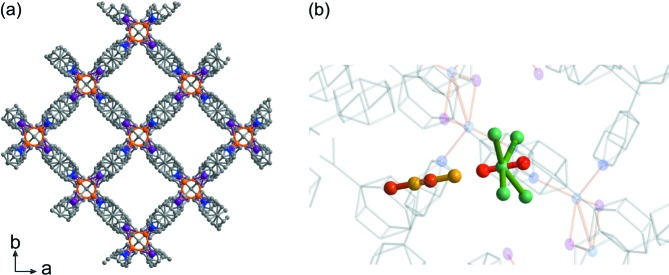
Crystal structures for (*a*) desolvated network **2** and (*b*) network **2** after sulfur encapsulation at 30 K. The stoichiometry of the structure is {[(Cu_2_I_2_)(C_45_H_32_N_4_)]·(S_2_)_0.975_}*_n_*. Part (*b*) shows a different view of disordered S_2_ in the one-dimensional channel with a ball-and-stick model: each coloured molecule corresponds to S_2_. Atom colouring: C grey, N blue, Cu orange, I purple, and S yellow, brown, red and green. H atoms have been omitted for clarity.

Kinetic trapping of sulfur gas in network **2** resulted in physisorbed S_2_ species only, with no evidence of S_3_. X-ray analysis at 30 K revealed that S_2_ physisorbed on two different sites of network **2**: (i) aligned in the one-dimensional channel of the structure and presenting severe disorder; and (ii) within small cavities adjacent to the Cu_2_I_2_ units (Fig. 4 ▸; see Fig. S8 in the supporting information for structure details). Only physisorption of S_2_ was observed on iodide sites in this network, because of steric hindrance around the iodide sites. The smaller size and linear shape of the one-dimensional channels suppress the conversion of linear S_2_ molecules into S_3_ species. This is an example of shape-selective trapping of a linear reactive intermediate. However, the S_2_ molecules existing adjacent to the Cu_2_I_2_ units have a weak interaction with iodide. These interactions come from charge transfer from iodide to sulfur, as shown by calculation (see Table S5 in the supporting information). This type of interaction is different from the Lewis acid–sulfide interaction shown in a sulfide-encapsulating Ni–MOF system (MOF = metal–organic framework; Zheng *et al.*, 2014 ▸). The existence of S_2_ in network **2** after sulfur encapsulation was further confirmed with microscopic Raman spectroscopy at room temperature; a new band appeared at 728 cm^−1^ (Fig. 3 ▸ *b*), which corresponds to S_2_ symmetric stretching (Barletta, 1971 ▸). S_2_ remained stable within network **2** up to 500 K (see Fig. S2 in the supporting information).

## 3. Conclusions   

We observed labile sulfur allotropes reacting in an interactive pore using X-ray diffraction. We found unexpected reactions of S_2_ on an interactive site: chemisorption, transformation of S_2_ into *cyclo*-S_3_, and bent-S_3_ species. On the basis of X-ray and vibrational analyses and theoretical calculations, we propose that the chemisorbed species is *cyclo*-S_3_^2+^ rather than neutral *cyclo*-S_3_. We also, for the first time, isolated S_2_ in a one-dimensional channel by kinetically suppressing further reactions. The method reported here provides a new means for future investigations of other labile reaction intermediates. Indeed, this method makes it possible to find out new reactions of sulfur allotropes.

## 4. Related literature   

The following references are cited in the supporting information for this article: Alecu *et al.* (2010 ▸), Allen (2002 ▸), Becke (1997 ▸), Chai & Head-Gordon (2008*a* ▸,*b* ▸), Frisch *et al.* (2009 ▸), Grimme (2006 ▸), Kozuch & Martin (2013 ▸), Sheldrick (1990 ▸) and Weigend & Ahlrichs (2005 ▸).

## Supplementary Material

Crystal structure: contains datablock(s) S_dimer, 250K_S_helical_initial, 300K_S_helical, 350k_S_helical, 300k_only_once. DOI: 10.1107/S2052252516008423/ed5008sup1.cif

Structure factors: contains datablock(s) S_dimer. DOI: 10.1107/S2052252516008423/ed5008S_dimersup2.hkl

Structure factors: contains datablock(s) 250K_S_helical_initial. DOI: 10.1107/S2052252516008423/ed5008250K_S_helical_initialsup3.hkl

Structure factors: contains datablock(s) 300K_S_helical. DOI: 10.1107/S2052252516008423/ed5008300K_S_helicalsup4.hkl

Structure factors: contains datablock(s) 350k_S_helical. DOI: 10.1107/S2052252516008423/ed5008350k_S_helicalsup5.hkl

Structure factors: contains datablock(s) 300k_only_once. DOI: 10.1107/S2052252516008423/ed5008300k_only_oncesup6.hkl

Supporting information. DOI: 10.1107/S2052252516008423/ed5008sup7.pdf

CCDC references: 1415696, 1415697, 1415698, 1415699, 1415700
