# Supplementary material for: Isolation and evolution of labile sulfur allotropes via kinetic encapsulation in interactive porous networks
Source: IUCrJ. 2016 Jun 8;3(Pt 4):232–6. doi: 10.1107/S2052252516008423 (PMC4937778; doi:10.1107/S2052252516008423)
Supplement: Supplementary file 7 [file m-03-00232-sup7.pdf]

IUCrJ

Volume 3 (2016)

Supporting information for article:

Isolation and evolution of labile sulfur allotropes *via* kinetic encapsulation in interactive porous networks

Hakuba Kitagawa, Hiroyoshi Ohtsu, Aurora J. Cruz-Cabeza and Masaki Kawano

Supporting information

S1. Experimental

S1.1. Materials and Methods

Chemical reagents were used as received from the vendors. Network isomer **1** and network isomer **2** were synthesized as previously reported.[S1] The diffraction data for small sulphur species encapsulating network **1** was collected on an ADSC Quantum-210 detector with a synchrotron radiation (λ = 0.800 or 0.700 Å) at 2D SMC beamline of the Pohang Accelerator Laboratory (PAL), Korea. The diffraction data for small sulphur species encapsulating network **2** was collected on a RIGAKU/MSC Mercury CCD X-ray diffractometer with a synchrotron radiation (λ = 0.6889 Å) at PF-AR (NW2A beamline) of the High Energy Accelerator Research Organization (KEK), Japan. The structures were solved by direct methods (SHELXS-97) and refined by full-matrix least squares calculations on F^2 (SHELXL-97 or SHELXL-2014) using the SHELX-TL program package.[S2] Raman spectra were acquired by an inVia Raman microscope (Renishaw) with 830 nm wavelength excitations to avoid strong emission from the network. IR spectra were recorded on a Varian 670-IR FT-IR spectrometer by the microscope method. Elemental analysis was performed on an Elementar vario MICRO cube at Technical Support Center in Pohang University of Science and Technology. Thermogravimetric (TG) analysis was carried out at a ramp rate of 10 K/min in a nitrogen flow (20 ml/min) with a Perkin-Elmer Pyris1 TGA.

S1.2. Kinetic trapping of small sulphur into network 1 and 2

S1.2.1. Kinetic trapping of sulphur-gas in network 1

A glass cup (*ca*. 1 mL) containing the desolvated crystals of **1** (*ca*. 2 mg) and a glass cup (*ca*. 2 mL) containing commercially available sulphur (cyclo-S_8) (*ca*. 50 mg) were put in a zig-zag shaped glass tube (inner diameter: 10 mm; length: 250 mm, ca. 12 mL) at different sites (Figure S1). After the glass tube was sealed under ~2.0 × 10^{-6} Torr, the sulphur site was heated by flame. The crystals changed its colour after a while. Brownish yellow crystals were collected.

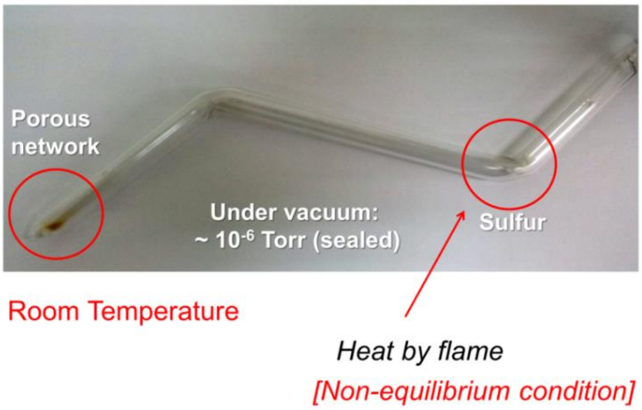

Figure S1 The set-up for kinetic trapping of sulphur-gas. In this sealed tube (~10-6 Torr), only the sulphur site was flamed by a hand burner (using butane gas).

S1.2.2. Kinetic trapping of sulphur-gas in network 2

A glass cup (*ca.* 1 mL) containing the desolvated crystals of **2** (*ca.* 2 mg) and a glass cup (*ca.* 2 mL) containing commercially available sulphur (cyclo-S_8) (*ca.* 50 mg) were put in a zig-zag shaped glass tube (inner diameter: 10 mm; length: 250 mm, *ca.* 12 mL) at different sites. After the glass tube was sealed under ~2.0 × 10^{-6} Torr, the sulphur site was heated by flame. Yellow crystals were collected.

S1.2.3. Elemental and Thermogravimetrical Analysis of sulphur-encapsulating networks 1 & 2

Direct measurement of elemental analysis (EA) was only possible for the elements C, H, N and S. CuS ash remained in the sample holder after the analysis. We calculated the ratio of C, H, N and S combined with the results of X-ray analysis and TG as follows. For network **1** after the exposure to sulphur gas, X-ray analysis suggested the component, $\{[(Cu_{1.88}I_{1.88})(C_{45}H_{32}N_4)]\bullet (S_3)_{0.74}\}$. However, we could see ~ 8 % of DMSO was escaped at 570 K corresponding to one DMSO in the pore which is invisible in X-ray analysis due to severe disorder of DMSO. So overall structure for network **1** after the exposure to sulphur gas is $\{[(Cu_{1.88}I_{1.88})(C_{45}H_{32}N_4)]\bullet DMSO\bullet (S_3)_{0.74}\}$. The calculated value of EA for network **1** after the encapsulation of sulphur species is coming from this formula. For network **2** after the exposure to sulphur gas, X-ray analysis suggested the component, $\{[(Cu_2I_2)(C_{45}H_{32}N_4)]\bullet (S_2)_{0.975}\}$. Because we did not see DMSO escape by TG, we calculated EA value using this formula.

The EA of the sample compositions is given in Table S1 and the calculated formulas are also given. The observed sulphur value is smaller than calculated value because small sulphur reacted with Cu to form CuS and remained as ash. Therefore, observed S value is corresponding to the amount of DMSO (2.82 % for network **1**, 0 % for network **2**).

Table S1 Elemental analysis for networks **1** and **2** after sulphur exposure (values in %).

	**Network 1 after sulphur exposure**	**Network 2 after sulphur exposure**
1st Measurement	C = 50.01<br>H = 3.465<br>N = 4.820<br>S = 2.855	C = 48.73<br>H = 3.125<br>N = 5.069<br>S = 0.503
2nd Measurement	C = 49.93<br>H = 3.554<br>N = 4.858<br>S = 2.844	C = 51.04<br>H = 3.252<br>N = 5.214<br>S = 0.493
Calculated	C = 49.69<br>H = 3.37<br>N = 4.93<br>S = 9.09 (2.82 from DMSO)	C = 50.41<br>H = 3.01<br>N = 5.23<br>S = 5.83
Formula	**$\{[(Cu_{1.88}I_{1.88})(C_{45}H_{32}N_4)]\bullet DMSO\bullet (S_3)_{0.74}\}$**	**$\{[(Cu_2I_2)(C_{45}H_{32}N_4)]\bullet (S_2)_{0.975}\}$**

The non-stoichiometric number for CuI comes from defect site which is consistent with X-ray analysis.

TG of networks **1** and **2** after sulphur encapsulation show network decomposition at 670 K, when the sample is transformed into CuS ash (Figure S2). Before that, in network **1**, there is some loss of weight around 500 K which is ascribed to residual DMSO (and DMS) species.

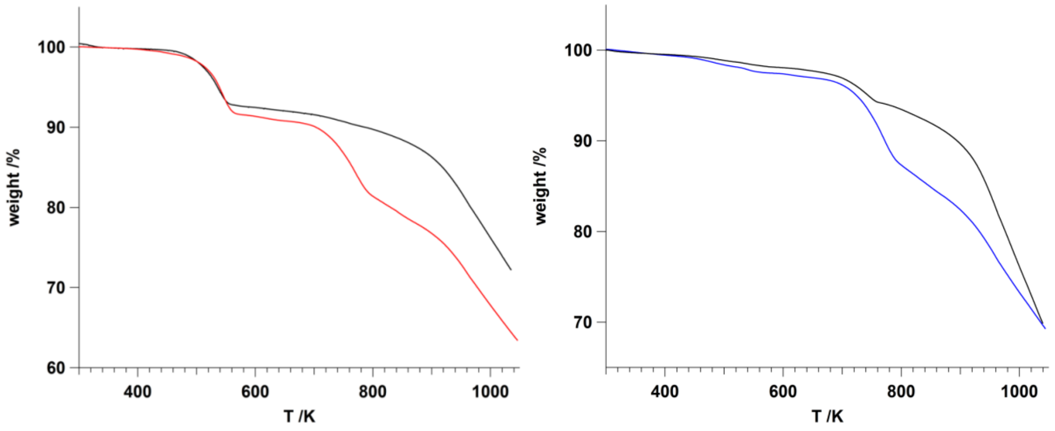

Figure S2 **(Left)** TG of network **1** desolvated (black) and after sulphur encapsulation (red). The weight loss at 480 K is due to residual DMSO. **(Right)** TG of network **2** desolvated (black) and after sulphur encapsulation (blue). Both networks decompose above 670 K resulting in CuS ash.

S1.3. Single crystal X-ray diffraction

After the kinetic trapping of the sulphur, single crystal X-ray diffraction was performed with various samples.

For network **1** samples, a single crystal was quickly picked up after sulphur exposure with a Litholoop and mounted on a goniometer immediately. After centering, full data sets (ω-scan) were collected at 250 K for *ca*. 30 min. Then, the crystal was heated to 300 K and after 5 min full data sets were collected at 300 K. The same procedure was repeated at 350 K and down to 250 K (Figure S3). We used only the Bragg diffractions for structure solution and data processing. We observed, however, diffuse scattering on the first measurement at 250 K (Figure S4). The entire cycle in Figure S3 was repeated several times and reproducibility of the results was confirmed with the electron density maps. However, we observed slight variations depending on the encapsulation time used and the cooling rates because of the kinetic nature of the trapping process.

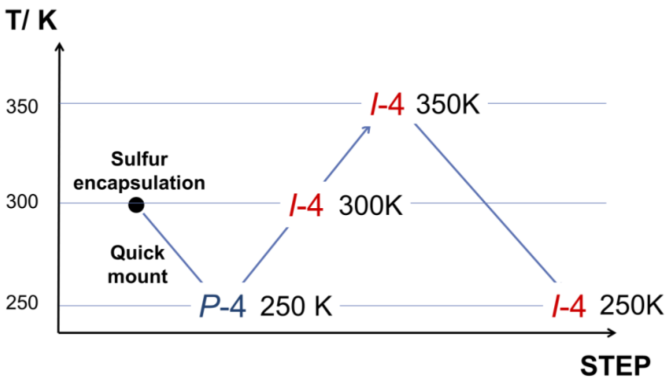

Figure S3 The X-ray diffraction experiments procedure after kinetic trapping of small sulphur to network **1**.

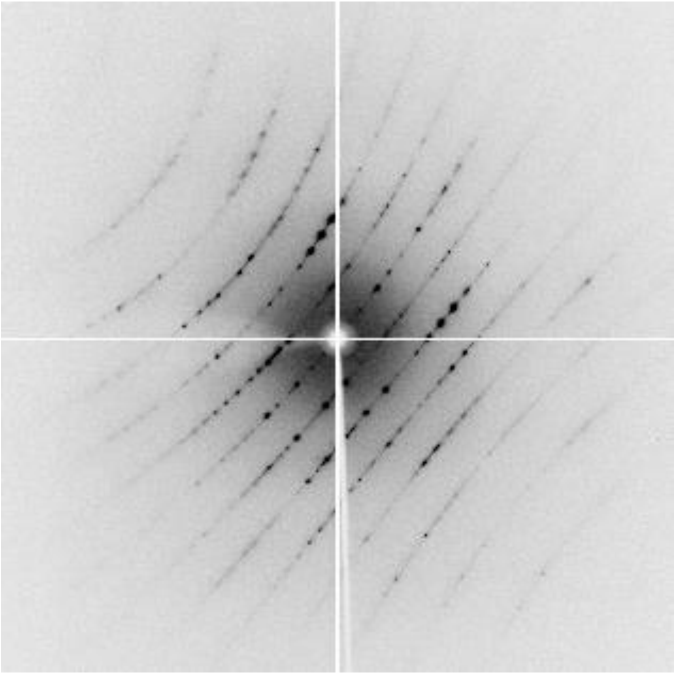

Figure S4 One of the diffraction images of small sulphur encapsulating network **1** right after encapsulation collected at 250 K. Many diffuse scatterings observed in **a*** × **b*** plane corresponds to the channel direction.

For network **2** samples, a single crystal was quickly collected after sulphur exposure and mounted on a goniometer. After centering, the full data sets (ω-scan) were collected at 30 K. Few diffuse scattering was observed in network **2** samples.

Structure solution details for modelling of sulphur species:

1) For chemisorbed sulphur species:

For making sulphur models, we did not use any restraints for chemisorbed sulphur species. We obtained excellent agreement between X-ray and theoretical models as described in the manuscript (see Figure 2).

2) For free sulphur species:

We made models of S_2 and S_3 on the basis of our previous result and reference results obtained by spectroscopy to softly restrain the bond and angle parameters. The bond length of S-S is in the range of 1.8 – 2.2 Å. We picked up such electron density pairs in the electron density map. Judging from the atomic displacement parameters and occupancy factors, we could make correct models for free S_2 and S_3 in a pore. At the final stage of refinement, we removed many restraints except the case where

Q peaks were too closely located which led to unstable convergence. We also could conclude that there was no S_4, S_5, S_6 in a channel on the basis of X-ray and FT-IR spectroscopic results.

Therefore, we did not make models of S_2 and S_3 randomly. Because it is not easy to figure out which electron densities are paired, we performed the same experiments many times using synchrotron source (PAL 2D). In addition, we also carefully checked our results using various methods like FT-IR, Raman, UV-vis and theoretical calculation as well as chemical common sense.

Regarding displacement parameters: Because network **1** showed phase transition at temperatures lower than 250 K, we had to measure X-ray data over 250 K. Naturally the displacement parameters became large for guest molecules of S_2 and S_3 species which are gas at an ambient condition. We observed kinetic state by X-rays. That means that what we observed is the averaged state of guest during reaction. Naturally this effect made the displacement parameters larger than normal. Therefore, we repeated the same synchrotron experiments several times to confirm reproducibility of electron density.

Crystallographic data for

(1) Kinetically trapped sulphur into network crystal 1

(1-1) measured at 250 K (initial stage):

$\{[(CuI)_{1.88}(C_{45}H_{32}N_4)]\bullet(S_2)_{0.63}\bullet(\text{bent-}S_3)_{0.58}\}_n$

$C_{45}H_{32}N_4S_{3.00}$ $Cu_{1.88}I_{1.88}$, M_r = 1083.02, crystal dimensions 0.76 × 0.05 × 0.05 mm^3, tetragonal, space group *P*-4, a = 26.7176(5) Å, c = 7.1942(2) Å, V = 5135.4(2) Å^3, Z = 4, ρ_{calcd} = 1.403 g cm^{-3}, μ = 27.07 cm^{-1}, λ = 0.8000 Å (synchrotron radiation), T = 250(2) K, 4892 unique reflections out of 7133 with $I > 2\sigma(I)$, 750 parameters, 1237 restraints, 2.714 < θ < 26.386 °, final R factors $R1$ = 0.0675 and w$R2$ =0.2346, GOF = 1.013. CCDC deposit number 1415697.

(1-2) measured at 300 K :

$\{[(CuI)_{1.88}(C_{45}H_{32}N_4)]\bullet(\text{cyclo-}S_3)_{0.62}\bullet(\text{bent-}S_3)_{0.39}\}_n$

$C_{45}H_{32}N_4S_{3.01}Cu_{1.88}I_{1.88}$, M_r = 1083.34 crystal dimensions 0.76 × 0.05 × 0.05 mm^3, tetragonal, space group *I*-4, a = 26.8758(4) Å, c = 7. 1689(1) Å, V = 5178.1(2) Å^3, Z = 4, ρ_{calcd} = 1.397 g cm^{-3}, μ = 27.15 cm^{-1}, λ = 0.8000 Å (synchrotron radiation), T = 300(2) K, 3852 unique reflections out of 4353 with $I > 2\sigma(I)$, 365 parameters, 397 restraints, 2.698 < θ < 28.077 °, final R factors $R1$ = 0.0389 and w$R2$ = 0.1182, GOF = 1.079. CCDC deposit number 1415698.

(1-3) measured at 350 K:

$\{[(CuI)_{1.88}(C_{45}H_{32}N_4)]\bullet(\text{bent-}S_3)_{1.00}\}_n$

$C_{45}H_{32}N_4S_{3.01}Cu_{1.88}I_{1.88}$, M_r = 1083.34, crystal dimensions 0.76 × 0.05 × 0.05 mm^3, tetragonal, space group *I*-4, *a* = 26.8987(5) Å, *c* = 7. 1788(1) Å, *V* = 5194.1(2) Å^3, *Z* = 4, ρ_{calcd} = 1.384 g cm^{-3}, μ = 26.76 cm^{-1}, λ = 0.8000 Å (synchrotron radiation), *T* = 350(2) K, 3278 unique reflections out of 3717 with *I* > 2σ(*I*), 338 parameters, 77 restraints, 2.695 < θ < 26.391 °, final *R* factors *R*1 = 0.0412 and w*R*2 = 0.1203, GOF = 1.053. CCDC deposit number 1415699.

(2) Kinetically trapped sulphur into network crystal 2:

$\{[(Cu_2I_2)(C_{45}H_{32}N_4)]\bullet(S_2)_{0.975}\}_n$

$C_{45}H_{32}Cu_2I_2N_4S_{1.95}$, M_r = 1070.94, crystal dimensions 0.05× 0. 03 × 0.03 mm^3, tetragonal, space group *I*4/*mcm*, *a* = 13.2563(2), *c* = 27.5616(7) Å, *V* = 4843.4(2) Å^3, *Z* = 4, ρ_{calcd} = 1.473 g cm^{-3}, μ = 19.75 cm^{-1}, λ = 0.6889 Å (synchrotron radiation), *T* = 30(5) K, 2025 unique reflections out of 3092 with *I* > 2σ(*I*), 140 parameters, 43 restraints, 2.11 < θ < 35.00 °, final *R* factors *R*1 = 0.0372 and w*R*2 = 0.0981, GOF = 0.752. CCDC deposit number 1415696.

(3) Kinetically trapped sulphur into network crystal 1 obtained only once measured at 300 K:

$\{[(CuI)_{1.85}(C_{45}H_{32}N_4)]\bullet(S_2)_{1.50}\}_n$

$C_{45}H_{32}N_4S_{3.00}Cu_{1.85}I_{1.85}$, M_r = 1077.31, crystal dimensions 0.15 × 0.08 × 0.08 mm^3, tetragonal, space group *I*-4, *a* = 26.8664(4) Å, *c* = 7.1717 (1) Å, *V* = 5176.5(2) Å^3, *Z* = 4, ρ_{calcd} = 1.390 g cm^{-3}, μ = 18.61 cm^{-1}, λ = 0.7000 Å (synchrotron radiation), *T* = 300(2) K, 4784 unique reflections out of 6115 with *I* > 2σ(*I*), 356 parameters, 100 restraints, 2.361 < θ < 29.511 °, final *R* factors *R*1 = 0.356 and w*R*2 = 0.1048, GOF = 0.993. CCDC deposit number 1415700.

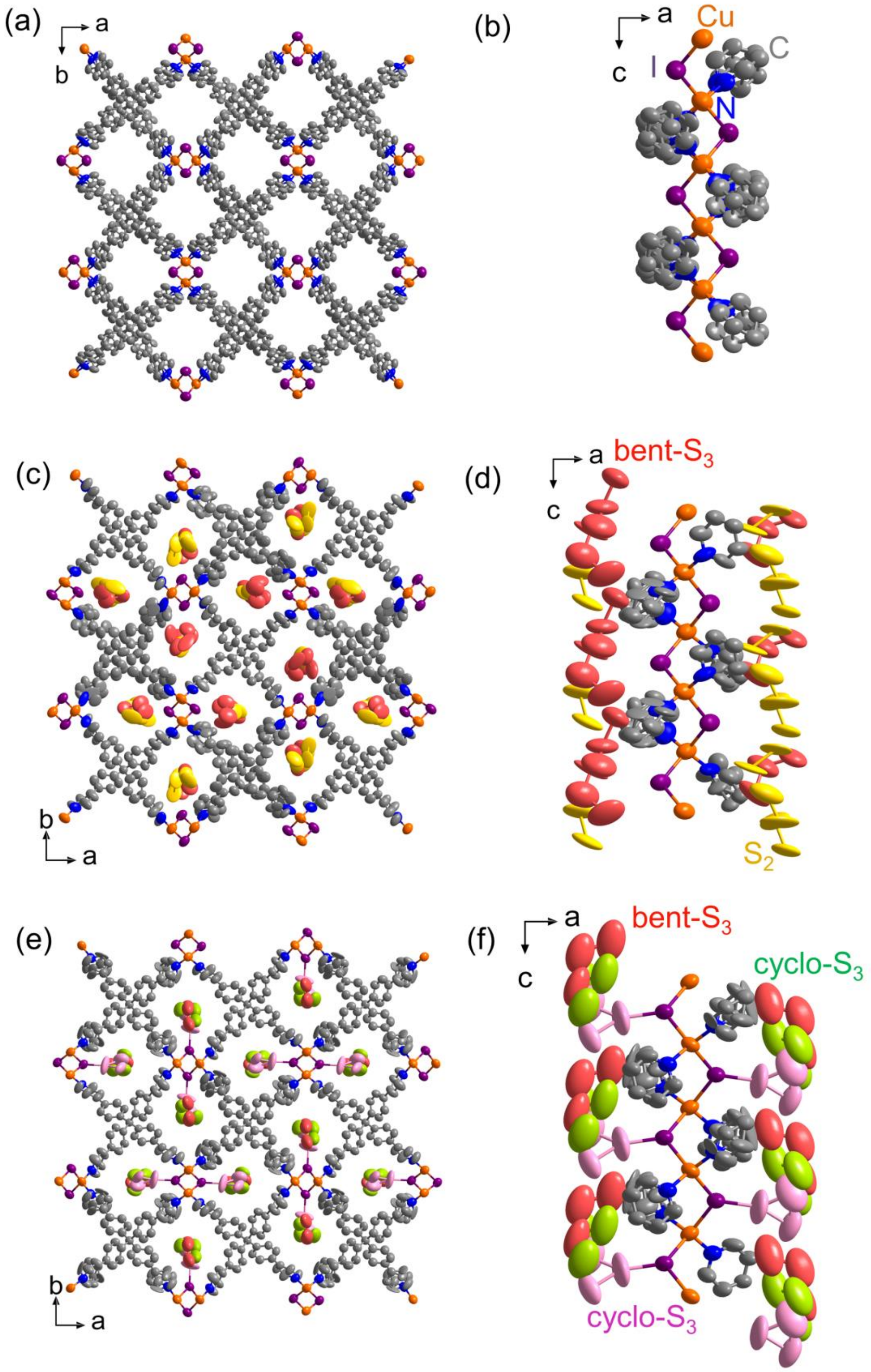

Figure S5 ORTEP plots (50% probability) for desolvated network **1** (a, b) and network **1** after sulphur encapsulation at 250K (c, d) and 300 K (e, f). The stoichiometries for the structures at 250 K and 300 K are $\{[(CuI)_{1.88}(C_{45}H_{32}N_4)]\bullet(S_2)_{0.63}\bullet(\text{bent-}S_3)_{0.58}\}_n$ and $\{[(CuI)_{1.88}(C_{45}H_{32}N_4)]\bullet(\text{cyclo-}S_3)_{0.62}\bullet(\text{bent-}S_3)_{0.39}\}_n$ respectively. Atoms colouring: C, grey; N, blue; Cu, orange; I, purple, S, yellow, red, green and pink (to specify the disordered S_2 and S_3 molecules). Hydrogen atoms are omitted for clarity.

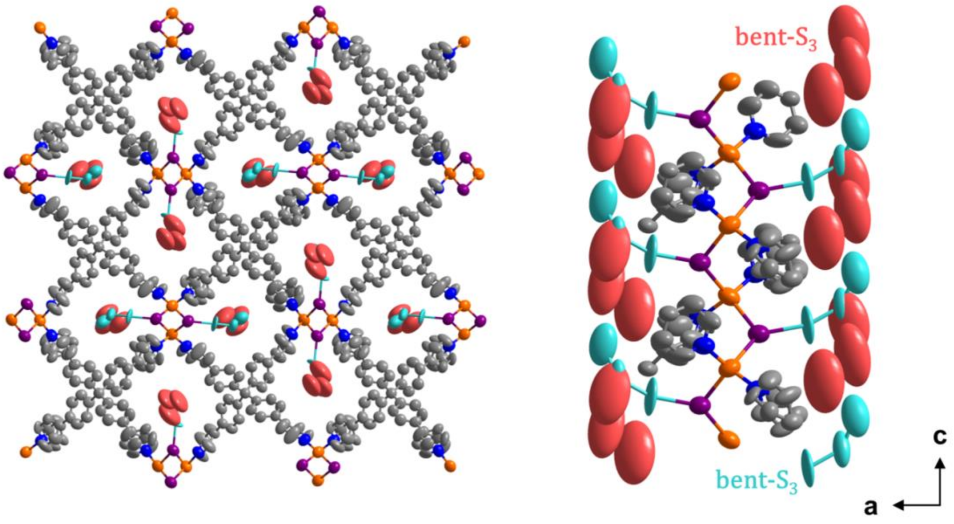

Figure S6 ORTEP drawing (50% probability) of sulphur encapsulating network **1** at 350K. Colours: C, grey; N, blue; Cu, orange; I, purple, S, red and cyan. Hydrogen atoms are omitted for clarity.

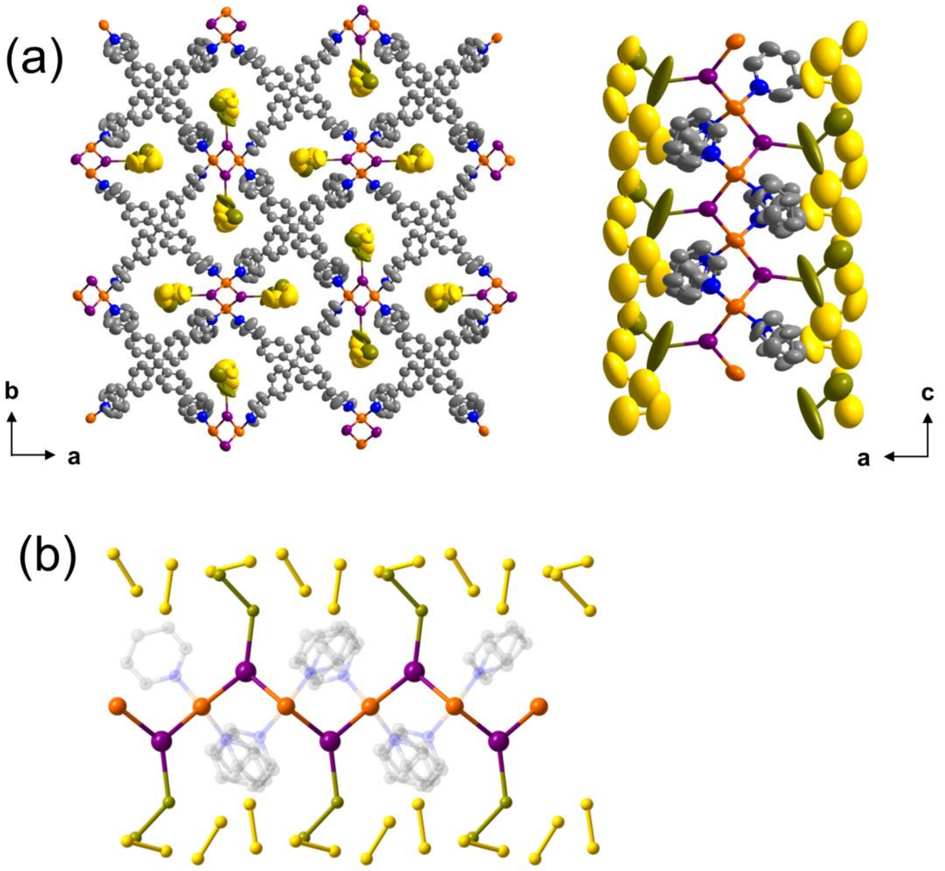

Figure S7 The crystal structure of small sulphur encapsulating network **1** which obtained one time at 300 K. The S_2 was physi- and chemi-sorbed in a channel. (A) ORTEP drawing (50% probability) of the S_2 encapsulating network **1**. (B) Ball and Stick model of disordered S_2. Colours: C, grey; N, blue; Cu, orange; I, purple, S, yellow and olive. Hydrogen atoms are omitted for clarity.

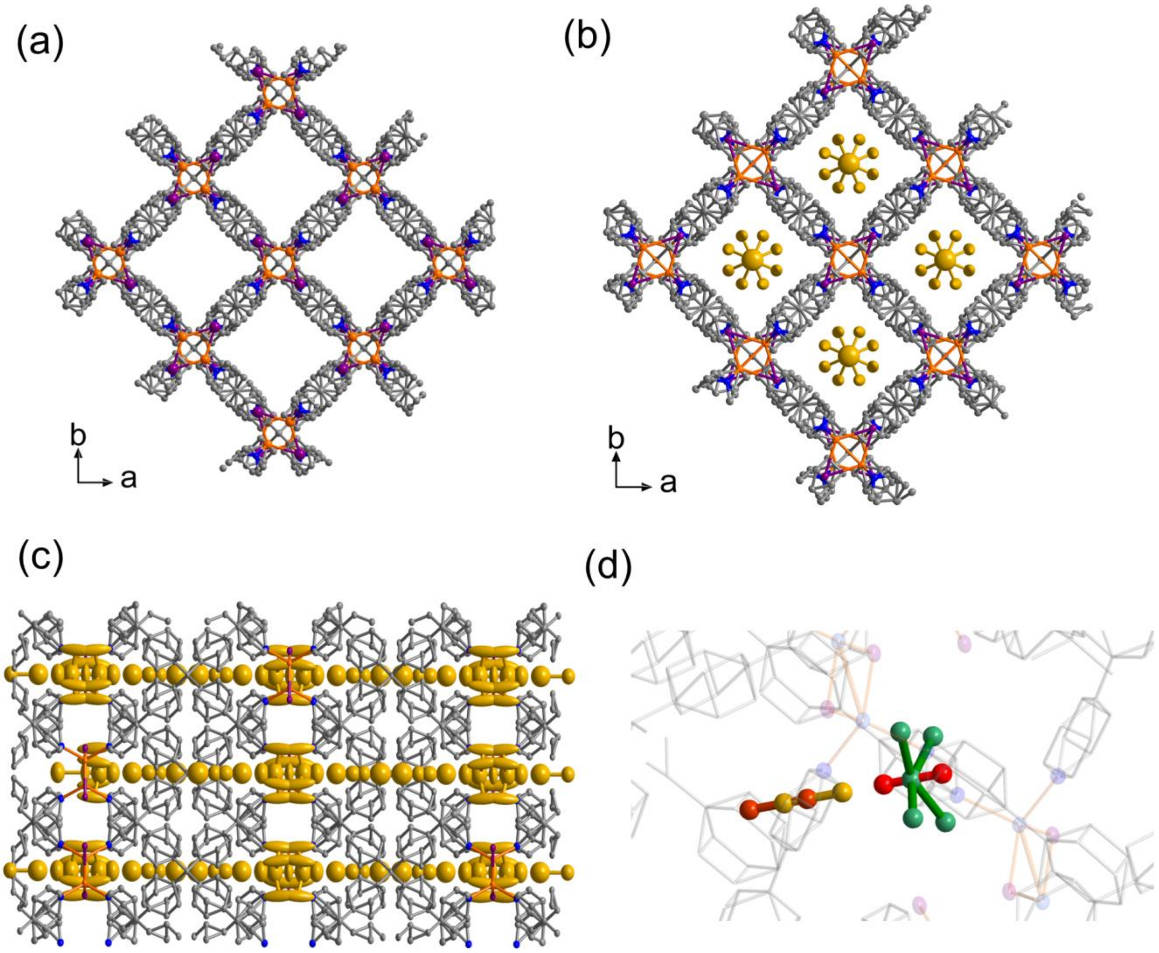

Figure S8 ORTEP plots (50% probability) for desolvated network **2** (a) and network **2** after sulphur encapsulation at 30 K (b and c). The stoichiometry of the 30 K structure is $\{[(Cu_2I_2)(C_{45}H_{32}N_4)]\bullet(S_2)_{0.975}\}_n$. (d) shows a different view of disordered S_2 in the 1D-channel with a Ball and Stick model: each coloured molecule corresponds to S_2. Atoms colouring: C, grey; N, blue; Cu, orange; I, purple, S, yellow, brown, red and green. Hydrogen atoms are omitted for clarity.

S1.4. Vibrational spectra measurements for small sulphur-encapsulating networks

Vibrational spectra for samples of networks **1** and **2** after sulphur exposure were recorded with microscopic Raman and IR spectroscopy. Because of apparatus limitations, we were only able to work at room temperature.

Figure S9 shows the Raman spectra of network **1** samples right after sulphur exposure (yellow) and 18 hours later (brown). We can see how the bands corresponding to cyclo-S_3 species (which were assigned with the aid of DFT calculations, see section 3) decrease with time.

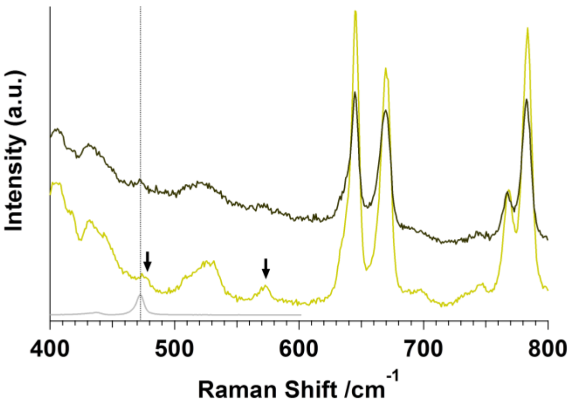

Figure S9 Raman spectra of network **1** immediately after sulphur exposure (yellow) and 18 hours later (brown). The grey line is the Raman spectrum of S_8. The arrows point to the vibrational bands of cyclo-S_3^{2+} which decrease significantly in the 18h-old sample.

Figure S10 shows the IR spectra of network **1** before and after sulphur encapsulation. A new band corresponding to bent-S_3 species is observed at ~680 cm^{-1} after sulphur encapsulation (see section 3).

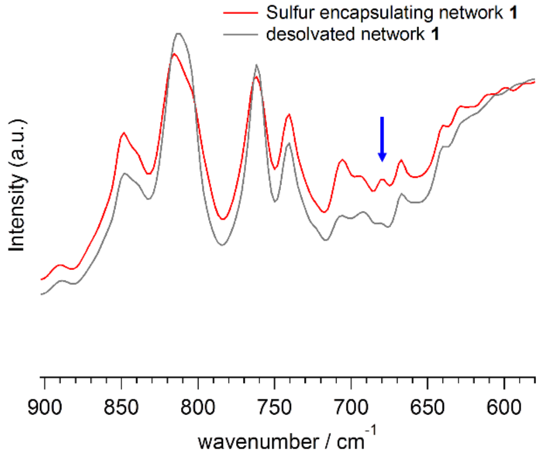

Figure S10 IR spectra of network **1** before (grey) and after (red) sulphur exposure. An arrow points the new band at ~680 cm^{-1} corresponding to bent-S_3.

We did not observe any change in the IR spectra of network **2** after sulphur exposure (Figure S11). This is because the only sulphur species present then is S_2 which has one vibrational mode which is IR inactive.

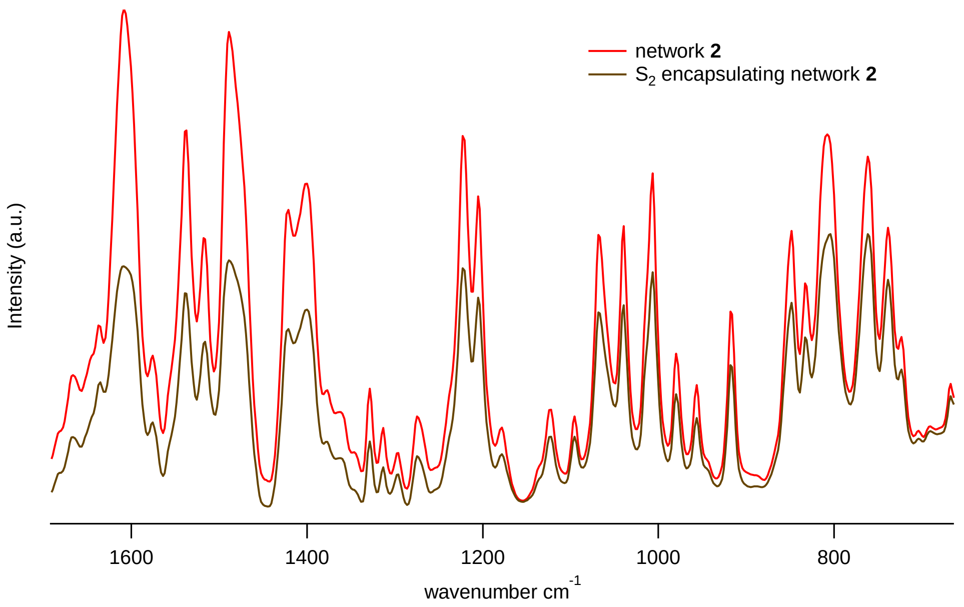

Figure S11 IR spectra of network **2** before (red) and after (black) sulphur exposure.

S2. Cambridge Structural Database Search for I…S interactions

S2.1. Methodology

The Cambridge Structural Database (CSD)[S3] was searched for I…S intramolecular bonds and intermolecular contacts with a distance less than the sum of the van der Waals radii plus 0.2 Å of each atom. No restrictions on the charge on the atoms were applied. The searches were performed to the entire CSD 5.34 (+ 3 updates). Only structures for which all heavy atoms coordinates had been determined were considered. This resulted in a total of 642 d(S…I) observations of both intra- and inter- molecular in the d(S…I) range between 2.35 Å and 3.65 Å.

S2.2. Distribution of I…S distances in the CSD

We searched the CSD for crystal structures containing interacting I…S atoms. This resulted in 642 observations of such distances, which were binned and plotted in the histogram in Figure S10.
We observe four main bands in the histogram in Figure S12. The first two bands are positioned at I…S distances of 2.6 and 2.8 Å. All these structures display an intramolecular I-S bond. Most of these observations correspond to molecules of I_2 forming a covalent bond with a sulphur atom in two type of environments: bonded to another atom (S=) or two other atoms (–S-).

The band positioned at 3.3 Å correspond to intramolecular bonds of catemeric type (-S-I-I-S-) or intermolecular bonds of the type I^-…S or I…S=. Beyond 3.4 Å all S…I bonds are of intermolecular nature.

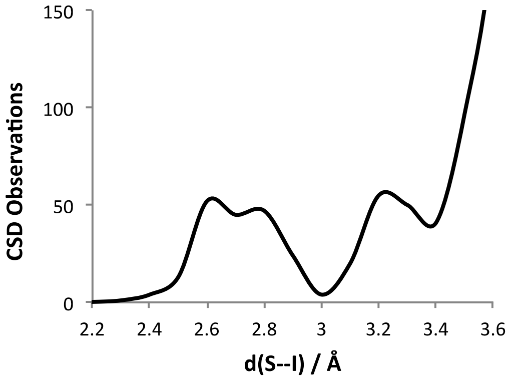

Figure S12 Histogram of S…I distances in the CSD (N = 642 observations for the presented distance range).

It is important to notice that, in all of these CSD examples, there tends to be an I_2 molecule or I^- ion interacting with an organic molecule containing sulphur. There are no examples in the CSD containing the neutral S_2 molecule and only one example containing the S_2^{2-} anion.
In summary, most intramolecular I…S bonds lie between 2.6 – 2.8 Å, most intermolecular I^-…S involving an I^- anion lie between 3.2 – 3.4 Å and the rest of neutral intermolecular I…S contacts tend to be larger than 3.4 Å. These CSD observations indicate that there is an affinity between I^- atoms to form strong intra- and/or inter- molecular bonds with sulphur containing molecules. Hence, our choice of networks **1** and **2** containing exposed I^- bridging atoms as potential traps for sulphur allotropes.

S3. Calculations of sulphur allotropes geometries and vibrational frequencies

S3.1. Methods

Geometry optimizations of the various sulphur allotropes in isolation and adsorbed on I^- anions in the gas-phase were performed with the code Gaussian09.[S4] We used the long-range corrected wB97XD DFT functional,[S5-S7] which includes the Grimme D2[S8] van der Waals corrections, with Def2-TZVPP basis sets[S9]. For simulating the Iodine atoms, effective core potentials were also used (Def2-TZVPP-ECP basis sets) which partly account for relativistic effects in heavy atoms. The model wB97XD/def2-TZVPP(-ECP) has given good results in the simulation of halogen bonds involving Iodine atoms, similar to the ones studied here.[S10] Geometry optimizations of dimers were performed in the gas-phase using tight convergence criteria. Some test calculations indicated that the basis-sets superposition errors (BSSE) with this model were small (< 1 kJ/mol). Hence, we decided not to include the BSSE corrections in the energies given. Interaction energies were calculated as the

difference between the dimer energy and the monomer energies. Calculations were performed with singlet and triplet electronic spins.

Frequency calculations were the performed on the optimized dimers and theoretical frequencies were scaled according to the factors derived in reference S11.

S3.2. Calculated and experimental geometries and their frequencies

We optimized the structures of S_2, S_3 and cyclo-S_3 allotropes with different charges and spins. The relative energies for the neutral species in singlet and triplet states are given in Table S2. The results of the optimized geometries and calculated and scaled frequencies are given in Table S3. We also present the same data as measured experimentally in Table S4. By comparing the optimized and the measured geometries, we are able to do some assignments.

Table S2 Relative stabilities (in kJ/mol) of the neutral species (q=0) in singlet (S=0) and triplet (S=1) states.

		**Isolated S allotropes**		**Allotropes adsorbed on I^-**	
	**q**	**Singlet (S=0)**	**Triplet (S=1)**	**Singlet (S=0)**	**Triplet (S=1)**
**S_2**	0	93.9	0	9.7	0.0
**S_3**	0	0	73.2	0	38.9
**cyclo-S_3**	0	0	-	-	-

From Table S2, we can see that for S_3 and cyclo-S_3, the singlet states are always favored. For S_2, however, the ground state is the triplet state. On adsorption onto I^-, however, S_2 in the singlet state becomes much more favourable energetically (see next section).

Table S3 Bond lengths (d) and angles (α) and frequencies (Freq) for the optimized S_2, S_3 and cyclo-S_3 species with various charges (q) and electronic spins (S) in the gas phase and adsorbed onto I^- anions. wB97XD/def2-TZVPP(-ECP) level of theory. The reported frequency corresponds to the S-S stretch which is Raman active for all but IR active only for the S_3 allotropes.

			Isolated S allotropes			Allotropes adsorbed on I^-			
	**q**	**S**	**d[S-S] (Å)**	**α[S-S-S] (°)**	**Freq (cm^{-1})**	**d[S-S] (Å)**	**α[S-S-S] (°)**	**d[S...I⁻] (Å)**	**Freq (cm^{-1})**
**S_2**	2+	0	1.749	-	928	1.849	-	2.340	743
	+	1/2	1.804	-	838	1.911	-	2.438	625
	0	0	1.884	-	719	**1.973**	**-**	**2.602**	**559**
	**0**	**1**	**1.884**	**-**	**720**	1.913	-	3.317	645
	-	1/2	2.004	-	572	-	-	-	-
	2-	0	2.206	-	410	-	-	-	-
**S_3**	2+	0	1.905	73	692	2.029, 1.853	115	2.316	723
	+	1/2	1.931	99	651	-	-	-	-
	**0**	**0**	**1.898**	**118**	**701**	**1.943, 1.977**	**116**	**2.721**	**594**
	-	1/2	1.986	115	565	-	-	-	-
	2-	0	2.117	114	441	-	-	-	-
**cyclo-S_3**	2+	0	1.931	60	692	**2.056**	**60**	**2.367**	**426, 591**
	+	1/2	1.996	60	643	-	-	-	-
	**0**	**0**	**2.073**	**60**	**448, 599**	-	-	-	-
	-	1/2	-	-	-	-	-	-	-
	2-	0	-	-	-	-	-	-	-

We can ignore the existence of S_4 or S_5 (and larger allotropes) from vibrational band (Meyer, 1976)

From Table S3 we can see the effect of charge and adsorption onto iodine atoms on the geometries and frequencies of the sulphur allotropes. Rows in blue correspond to the species that were identified during experiment and which are given in Table S4.

Table S4 Bond lengths (d), angles (α) and frequencies measured by X-rays and Raman spectroscopy. According the geometries, an assignation of the species is made after comparison with Table S2.

Structure	**T (K)**	**X-ray**	**d[S-S] (Å)**	**α[S-S-S] (°)**	**d[I-S] (Å)**	**Raman* (at 300 K)**	**Assignation**
*Network **2***	30	S_2	1.847 (1.863)	-	-	728	S_2 (triplet) in pore
*Network **1***	250	S_2	1.896	-	3.891	-	S_2 (triplet) in pore

		S_3 bent	1.924, 1.948	112	3.985	-	bent-S_3 (singlet) in pore
Network ***1***	300	S_3 bent<br>cyclo-S_3 chemi<br>cycloS_3 phys	1.898, 1.905<br>2.064<br>2.138	118<br>60<br>60	7.893<br>2.467<br>3.831	728<br>474, 573<br>474, 573	bent-S_3 (singlet) in pore<br>cycloS_3^{2+}-I^- (singlet) chemi.<br>cycloS_3 (singlet) in pore
Network ***1***	300	S_2 chemi.	2.232	-	2.613	-	S_2-I^- (singlet) chemi
Network ***1***	350	S_3	1.925(40)	117	4.061	-	bent-S_3 (singlet) in pore

*See Raman spectra in Figs. 3a and 3b of the manuscript.

Table S4 presents the geometries of the different allotropes as determined by X-ray diffraction at different temperatures. By comparing these geometries with the optimized ones in Table S3 we were able to make assignations on the species. There is generally a good agreement between the experimental geometries and those computed with DFT. The two new Raman bands observed upon sulphur encapsulation in network **1** (see Figure. 3) at 475, 573 cm^{-1} confirmed the existence of the cyclo-S_3 species neutral (448 and 599 cm-1) and with a 2+ charge chemisorbed on I^- (426 and 590 cm^{-1}) as observed by X-rays too.

S3.3. Physisorption and chemisorption of S_2 onto iodide atoms

Because we observed physisorbed as well as chemisorbed species of S_2 onto iodide atoms, we proceeded to evaluate the energetics of this process together with the electronic spin changes.

In the triplet electronic state (Table S5 and Figure S13), an S_2 molecule only physisorbs onto an I^- anion. The interaction energy of this intermolecular interaction is -29.2 kJ/mol and the bond order for the S…I interaction is just 0.1294. There is no covalent bond forming, just an intermolecular interaction of strength similar to a halogen bond. The interaction involves some charge transfer from the I^- atom (which in isolation has a charge of -1 but in the dimer of -0.786) to the furthest lying Sulphur atom mostly (-0.231, see Table S5). The equilibrium distance for this interaction is 3.317 Å adopting a bent geometry (with an I…S-S angle of 127.5 °).

In the singlet electronic state (Table S5 and Figure S13), however, the S_2 molecule chemisorbs onto an I^- anion. There is a covalent bond forming, the resulting I-S bond having a bond order of 0.9293. The chemisorbed geometry is also bent, with an I-S-S angle of 112.5 °. There is considerable charge transfer from the I- anion to the sulphur atoms. The I-S-S Mulliken charges are -0.372, -0.144 and -0.484 respectively. The far lying sulphur atom becomes the most negatively charged. The bond distance is 2.602 Å. There is clearly an intramolecular bond forming between the Iodine atom and the Sulphur atom. Paradoxically, however, the total energy gain in forming this intramolecular bond is just -19.4 kJ/mol, almost 10 kJ/mol less stabilizing than physisorption.

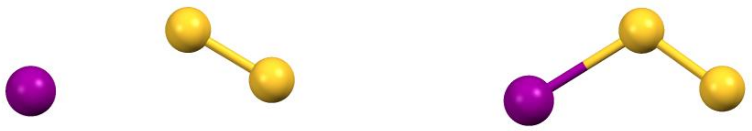

Figure S13 Optimized I^{-}…S_2 dimer in the triplet (left) and the singlet (right) spin states.

Table S5 Stability and key structural parameters for the optimized I^{-}…S_2 complex in the triplet and singlet spin

	I^{-}…S_2	
**Spin**	Triplet	Singlet
**Nature**	Physisorption	Chemisorption
**d(I-S) in Å**	3.317	2.602
**Wiber I-S bond order**	0.1943	0.9293
**d(S-S) in Å**	1.913	1.973
**Angle(I-S-S) in °**	127.5	112.5
**I-S-S Mulliken charges**	-0.786 0.017 -0.231	-0.372 -0.144 -0.484
**Bond Nature**	Intermolecular	Intramolecular
**Bond Energy (kJ/mol)**	-29.2	-19.4

Chemisorption is energetically less favored than physisorption in this system. The reason for this derives from the spin change (from triplet to singlet) needed for the chemisorption. The stabilities of the various (isolated non-interacting and interacting) systems are presented schematically in Figure S14.

The triplet spin state of the S_2 molecule is considerably more stable than the singlet state, by almost 94 kJ/mol. Hence, in isolation, the S_2 molecule in its ground state exists as a triplet. In the presence of the I^{-} anion, both the singlet chemisorbed and the triplet physisorbed have similar energies. They differ only by 10 kJ/mol. Respect to the isolated triplet state, the singlet chemisorbed S_2 is only 19 kJ/mol more stable. However, respect to the isolated singlet state, it is 113 kJ/mol more stable. This is indeed in the order of a covalent bond. This explains why, in this system, the chemisorbed species are, overall (because of the included spin energy), more weakly bound than the physisorbed species.
Finally, we studied the reaction pathway from a physisorbed triplet S_2 to a chemisorbed singlet S_2. As we can see from Figure S15, the physisorption to chemisorption process has an energy barrier of 15 kJ/mol. The transition state is approximately located a I…S distance of around 2.8 Å.

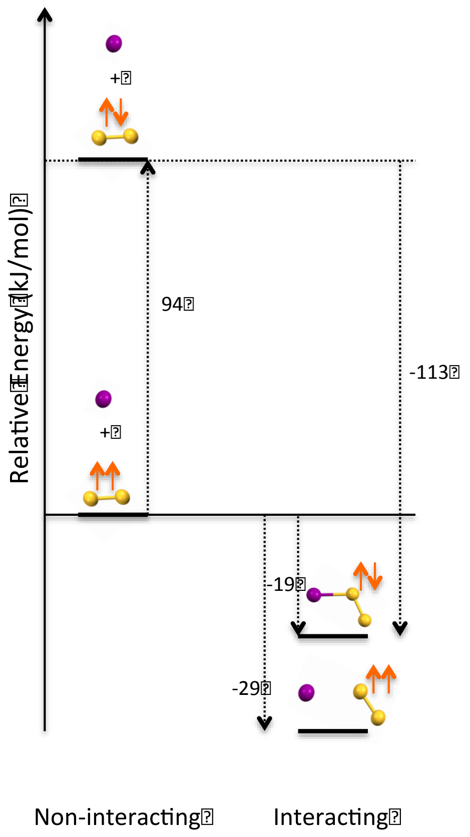

Figure S14 Schematic representation of the stability of the interacting $I^{-}\ldots S_2$ with respect to the isolated species and the singlet and triplet spins.

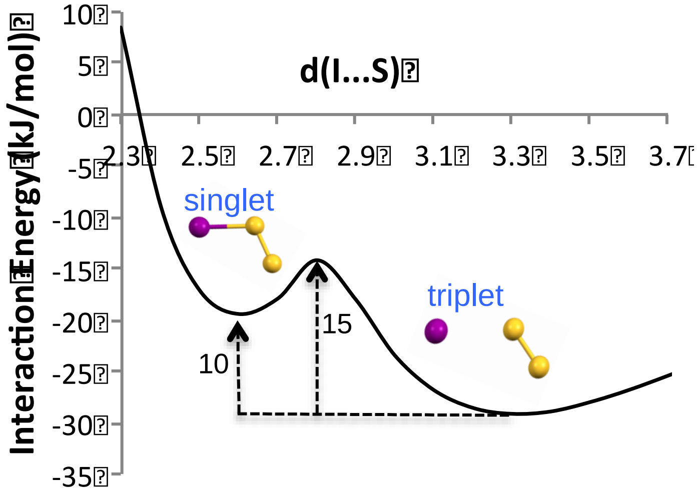

Figure S15 Potential energy curve of the physisorption to chemisorption process of a molecule of S_2 onto an I^{-} anion in the gas-phase.

S4. Possible reaction mechanisms taking place in network 1

There are two possible mechanisms as shown below:

(1) Direct conversion

The direct conversion of small sulphur species:

3 S_2 → 2 cyclo-S_3 → 2 cyclo-S_3^{2+} → 2 bent-S_3

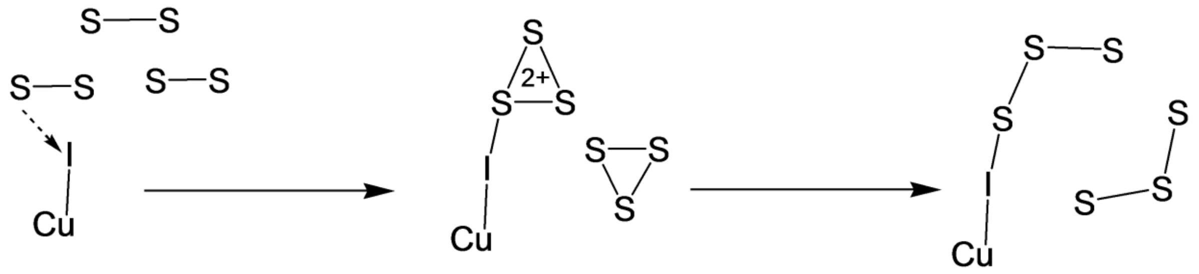

This reaction may be catalyzed by interactive iodide sites, otherwise this reaction may not proceed. The stabilization of S_3 species by iodide might be the driving force of this reaction.

Cyclo-S_3 was further oxidized by other sulphur species to produce H_2S_n species because a pore includes water (see Figure S16). We observed new bands typical of H-S stretches with IR spectroscopy at frequencies at 2225 cm^{-1} (see Figure S17).

(2) Mechanism involving DMSO

From elemental analysis we also know that there is about one molecule of DMSO per every two network iodides, so a 0.5:1 DMSO:I^- stoichiometry. Hence that the first step in the mechanism of S_2 gas transformation could be the reduction of DMSO and oxidation of sulphur gas (equation S1) to S_2O. S_2O is then known to decompose into S_3 and SO_2 (equation S2). The free energies of these reactions were calculated to be exothermic with DFT calculations.

S_2 + DMSO → S_2O + Me-S-Me ΔG = -56 kJ/mol   (S1)

S_2O → 0.5 S_3 + 0.5 SO_2 ΔG = -23 kJ/mol   (S2)

In this case, we suspect then that SO_2 gets further oxidized to SO_3 that reacts with water in a pore (Figure S16), resulting in an acidic environment. In this environment, we suspect that S_3 will oxidize into cyclo-S_3^{2+} with other sulphur molecules accepting electrons and protons resulting into H_2S_n species. We observed new bands typical of H-S stretches with IR spectroscopy at frequencies at 2225 cm^{-1} (see Figure S17).

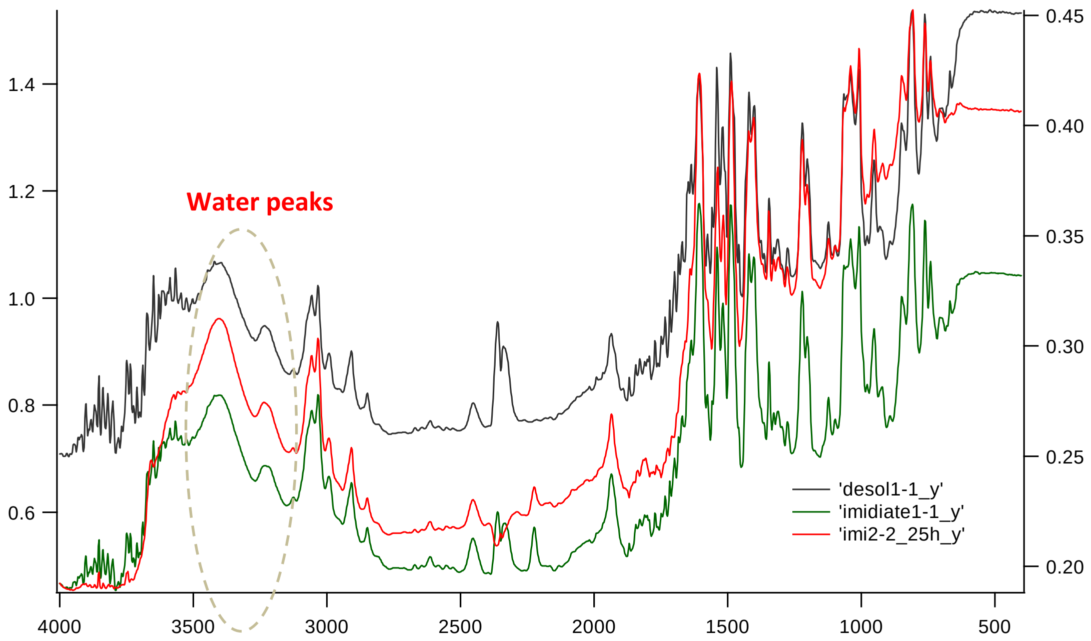

Figure S16 IR (black: desolvated network **1**, green: right after sulphur encapsulation, red: 25h after sulphur encapsulation) shows the presence of water in network **1**.

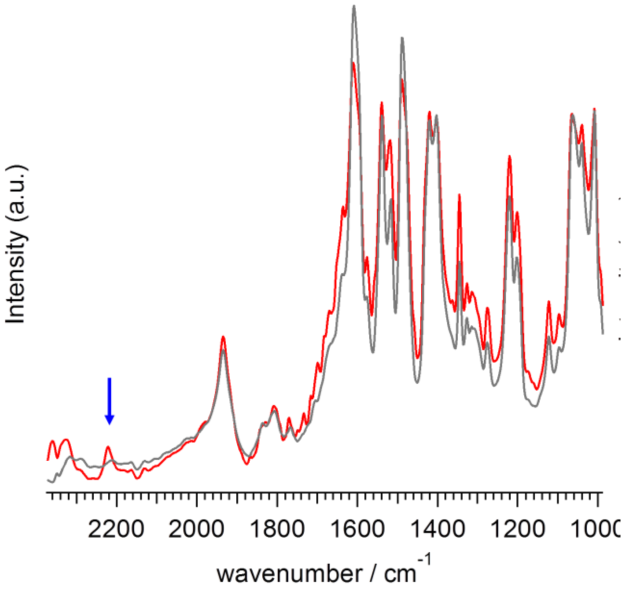

Figure S17 IR spectra of before (grey) and after (red) sulphur encapsulation to network **1**. The arrow shows the new bands at ~2225 cm^{-1} possibly due to S-H stretches.

S5. References for Supporting Information

(S1) Kitagawa, H., Ohtsu, H. & Kawano, M. (2013). *Angew. Chem. Int. Ed.*, **52**, 12395–12399.
(S2) Sheldrick, G. M. (1990). *Acta Crystallogr.* **A46**, 467-473.
(S3) Allen, F. H. (2002). *Acta Crystallogr*., **58B,** 380–388.
(S4) Gaussian 09, Revision D.01, M. J. Frisch, G. W. Trucks, H. B. Schlegel, G. E. Scuseria, M. A. Robb, J. R. Cheeseman, G. Scalmani, V. Barone, B. Mennucci, G. A. Petersson, H. Nakatsuji, M. Caricato, X. Li, H. P. Hratchian, A. F. Izmaylov, J. Bloino, G. Zheng, J. L. Sonnenberg, M. Hada, M. Ehara, K. Toyota, R. Fukuda, J. Hasegawa, M. Ishida, T. Nakajima, Y. Honda, O. Kitao, H. Nakai, T. Vreven, J. A. Montgomery, Jr., J. E. Peralta, F. Ogliaro, M. Bearpark, J. J. Heyd, E. Brothers, K. N. Kudin, V. N. Staroverov, R. Kobayashi, J. Normand, K. Raghavachari, A. Rendell, J. C. Burant, S. S. Iyengar, J. Tomasi, M. Cossi, N. Rega, J. M. Millam, M. Klene, J. E. Knox, J. B. Cross, V. Bakken, C. Adamo, J. Jaramillo, R. Gomperts, R. E. Stratmann, O. Yazyev, A. J. Austin, R. Cammi, C. Pomelli, J. W. Ochterski, R. L. Martin, K. Morokuma, V. G. Zakrzewski, G. A. Voth, P. Salvador, J. J. Dannenberg, S. Dapprich, A. D. Daniels, Ö. Farkas, J. B. Foresman, J. V. Ortiz, J. Cioslowski, and D. J. Fox, Gaussian, Inc., Wallingford CT, 2009.
(S5) Becke, A. D. (1997). *J. Chem. Phys.*, **107**, 8554.
(S6) Chai, J. –D. & Head-Gordon, M. (2008). *Chem. Phys.*,**128**, 084106.
(S7) Chai, J. –D. & Head-Gordon, M. (2008). *Phys. Chem. Chem. Phys*., **10**, 6615.
(S8) Grimme, S. (2006). *J. Comp. Chem.*, **27,** 1787-1799.
(S9) Weigend, F. & Ahlrichs, R. (2005). *Phys. Chem. Chem. Phys.*, **7,** 3297.
(S10) Kozuch, S. & Martin, J. M. (2013). *J. Chem. Theory Comput.*, ***9***, 1918–1931.
(S11) Alecu, I. M., Zheng, J., Zhao, Y. & Truhlar, D. G. (2010). *J. Chem. Theory Comput.*, **6**, 2872-2887.
